# Investigation into the effect of high concentrations of volatile fatty acids in anaerobic digestion on methanogenic communities

**DOI:** 10.1016/j.wasman.2014.07.020

**Published:** 2014-11

**Authors:** Ingrid H. Franke-Whittle, Andreas Walter, Christian Ebner, Heribert Insam

**Affiliations:** a Institut für Mikrobiologie, Universität Innsbruck, Technikerstraße 25, 6020 Innsbruck, Austria; b Abwasserverband Zirl und Umgebung, Meilbrunnen 5, 6170 Zirl, Austria

**Keywords:** Volatile fatty acids (VFAs), ANAEROCHIP, Real-time PCR, Methanogens, Anaerobic digestion (AD)

## Abstract

A study was conducted to determine whether differences in the levels of volatile fatty acids (VFAs) in anaerobic digester plants could result in variations in the indigenous methanogenic communities. Two digesters (one operated under mesophilic conditions, the other under thermophilic conditions) were monitored, and sampled at points where VFA levels were high, as well as when VFA levels were low. Physical and chemical parameters were measured, and the methanogenic diversity was screened using the phylogenetic microarray ANAEROCHIP. In addition, real-time PCR was used to quantify the presence of the different methanogenic genera in the sludge samples. Array results indicated that the archaeal communities in the different reactors were stable, and that changes in the VFA levels of the anaerobic digesters did not greatly alter the dominating methanogenic organisms. In contrast, the two digesters were found to harbour different dominating methanogenic communities, which appeared to remain stable over time. Real-time PCR results were inline with those of microarray analysis indicating only minimal changes in methanogen numbers during periods of high VFAs, however, revealed a greater diversity in methanogens than found with the array.

## Highlights

• Different methanogenic communities in mesophilic and thermophilic reactors.
• High VFA levels do not cause major changes in archaeal communities.
• Real-time PCR indicated greater diversity than ANAEROCHIP microarray.

## 1 Introduction

The anaerobic digestion (AD) of organic wastes is a sustainable waste management strategy that is gaining significance due to the increasing costs of fossil fuels and the need to mitigate anthropogenic global warming. Biogas production from various types of raw materials (e.g. manure, sewage sludge, food waste) has been shown to be a source of renewable energy that can occur sustainably in many different countries around the world (Bond and Templeton, 2011). The process produces a sludge of agricultural value, as well as biogas, which can be used to generate electricity and heat (Lastella et al., 2002, Insam and Wett, 2008). The efficient conversion of organic matter to methane in an anaerobic digester is dependent on the mutual and syntrophic interactions of functionally distinct microorganisms (Akuzawa et al., 2011). However, despite a continuously increasing interest and popularity of AD, the process remains inefficient and enigmatic. This is because of a lack of knowledge linking microbial community content, dynamics, and activity with reactor performance (Lee et al., 2009, Pycke et al., 2011).

The initial step in the AD process is the hydrolysis of the organic materials. During this step, macromolecules, such as proteins, carbohydrates and fats, are broken down to amino acids, sugars and fatty acids, respectively, by bacteria (Nielsen et al., 2007) and fungi (Leis et al., 2014). In the second stage of the process, acidogenic bacteria convert sugars, amino acids, and fatty acids to organic acids, alcohols and ketones, acetate, CO_2_, and H_2_. Acetogenic bacteria then convert fatty acids and alcohols into acetate, H_2_ and CO_2_, products used by methanogenic archaea to form biogas (typically 60% methane, 38% carbon dioxide and 2% trace gases). Archaea thus hold the key position in the methanisation. Methane can be produced by both acetotrophic and hydrogenotrophic methanogens, and differences in environmental conditions as well as reactor operating conditions (pH, temperature, hydraulic retention time, input material) have been reported to affect the composition of these communities (Demirel and Scherer, 2008, Akuzawa et al., 2011, Kim et al., 2013). Because the degradation phases are all closely connected with each other, an imbalance between the bacterial and archaeal communities can cause a deterioration in reactor performance, and thus changes in the amount of methane produced (Demirel and Yenigün, 2002, Rastogi et al., 2008, Akuzawa et al., 2011).

Reactor acidification through reactor overload is one of the most common reasons for process deterioration in anaerobic digesters (Akuzawa et al., 2011). This occurs because of a build-up of volatile fatty acids (VFAs) which are produced by acidogenic and acetogenic bacteria, and reflects a kinetic uncoupling between the acid producers and consumers (Ahring, 1995). High VFA concentrations cause pH values to decrease, and result in toxic conditions in the reactor. In anaerobic digesters with low buffering capacity, pH, partial alkalinity and VFAs are reliable indicators for process imbalance, however, in highly buffered systems, pH changes can be small, even when the process is extremely stressed, and only VFAs can be considered reliable for process monitoring (Murto et al., 2004). Various VFAs exist in ADs, and they have different and co-operative effects on bacteria and archaea. Wang et al. (2009) reported that acetic acid and butyric acid concentrations of 2400 and 1800 mg L^−1^, respectively, resulted in no significant inhibition of the activity of methanogens, while a propionic acid concentration of 900 mg L^−1^ resulted in significant inhibition of the methanogens. Opinions vary regarding which VFA is the best indicator for impending reactor failure, with different authors suggesting i-butyric, i-valeric, propionic acid, or the ratio of propionic:acetic acid as the most appropriate indicator (Boe, 2006). Nonetheless, it does not appear to be possible to define VFA levels to indicate the state of an anaerobic process, as different systems have their own levels of VFAs that can be considered ‘normal’ for the reactor, and conditions that cause instability in one reactor do not cause problems in another reactor (Angelidaki et al., 1993).

Other chemical compounds which are known to cause toxic effects in biogas reactors and can lead to a complete failure of methanogenesis are ammonia and hydrogen sulfide (Kayhanian, 1994, Chen et al., 2008), as well as accumulations of hydrogen and acetate, excess tannins, salts and heavy metals (Karri et al., 2006, Panyadee et al., 2013).

The aim of this study was to investigate the hypothesis that changes in the VFA levels of anaerobic digester plants can influence indigenous methanogenic communities. Archaeal communities present in two different anaerobic digesters were monitored using the ANAEROCHIP microarray. This microarray, which targets the 16S rRNA gene, offers the possibility to analyse an entire array of methanogens concerning their presence or absence in a particular sludge sample in a single experiment (Franke-Whittle et al., 2009a). Methanogens detected using the microarray were quantified using real-time PCR to determine exact numbers.

## 2 Materials and methods

### 2.1 Sampling of sludge from biogas producing reactors

Anaerobic sludges were collected from two AD plants (Inzing and Neustift) in Tirol, Austria, at various times (May 18, 2009; August 5, 2009; September 16, 2009 and October 27, 2009; for Neustift reactor, no samples for the August sampling were available). The Inzing reactor (I) was run under mesophilic conditions and had a reactor volume of 173 m^3^, an organic loading rate (OLR) of 2.8 kg VS m^−3^ d^−1^, a hydraulic retention time (HRT) of 57 d and a combined heat and power plant (CHP) with 20 kW electrical performance. The Neustift reactor (N) was run under thermophilic conditions, had a reactor volume of 110 m^3^, an OLR of 5.2 kg VS m^−3^ d^−1^, a HRT of 26 d and a CHP with 25 kW electrical performance. Both plants produced sludges that were used for agricultural purposes.

Information on the AD plants is listed in Table 1. Three bulked samples (each about 0.5 L) were collected from the reactors through the sampling ports.

**Table 1:** Reactor sampling dates, operational details and input materials.

Plant	Sampling date	Sample name	Operation temperature	Input material
Inzing	18.05.2009	I1	37–38 °C	Cow manure (46%), corn silage (36%), vegetable waste (9%), potato (9%)
	05.08.2009	I2	37–38 °C	Cow manure (46%), corn silage (36%), vegetable waste (9%), potato (9%)
	16.09.2009	I3*	37–38 °C	Cow manure (46%), corn silage (36%), vegetable waste (9%), potato (9%)
	27.10.2009	I4	37–38 °C	Cow manure (46%), corn silage (36%), vegetable waste (9%), potato (9%)

Neustift	18.05.2009	N1*	55 °C	Cow manure (52%), food waste (48%)
	16.09.2009	N3	55 °C	Cow manure (52%), food waste (48%)
	27.10.2009	N4*	55 °C	Cow manure (52%), food waste (48%)

*Note*: Vegetable waste refer to wastes obtained from the field after harvesting vegetables. Food waste refers to household kitchen waste. Low quality potatoes not fit for consumption included in Inzing reactor.

* High VFA samples.

### 2.2 Physical–chemical parameters

Temperature and biogas production were measured online in the reactors, and gas quality (CH_4_ [%], CO_2_ [%]) of the reactor was analysed with a portable Biogas Check BM 2000 instrument (Geotechnical Instruments, Warwickshire, UK). pH and electrical conductivity (EC) were measured in sludge samples at the time of collection using a portable multi-parameter meter Multi 340i (WTW, Weilheim, Germany). Total solids (TS) were calculated as the amount of solids remaining after oven-drying the samples overnight (105 °C). Volatile solids (VS) were calculated as the loss of weight after igniting the oven-dried residue at 550 °C for 5 h. Sample preparation for HPLC analysis was performed using dialysis and the method of Wagner et al. (2012). Following sample collection, a dialysis tube filled with 10 ml of distilled water was submerged into the liquid sample. The bottle was shaken three times and stored at 4 °C overnight in order to reach a total equilibrium in the dialysate. The tubing was removed, washed with a minimum volume of distilled water and opened. Dialysate (0.5 ml) was subjected to high performance liquid chromatography (HPLC) analysis on an Aminex HPX-87H column (Bio-Rad, Hercules, USA). A 5 mM H_2_SO_4_ mobile phase run at 0.7 ml min^−1^ and a detection wavelength of 210 nm were used. The detection limit for VFAs was >1 mmol l^−1^. Ammonium nitrogen (NH_4_–N) was measured photometrically after appropriate dilution with distilled water using the colorimetric tube test from Macherey-Nagel (Düren, Germany). NH_3_–N was calculated from NH_4_–N concentrations according to the formula of Calli et al. (2005). The PASW-SPSS 17.0 software was used to determine correlations between physical–chemical parameters and methanogenic genera present in sludges.

### 2.3 DNA extraction

The PowerSoil DNA Isolation Kit (MO BIO Laboratories, Carlsbad, California) was used to extract genomic DNA from sludge samples according to the instructions of the manufacturer, with the exception that the sample after being subjected to lysis buffers and mixing was exposed to three freeze–thaw cycles (30 min at −80 °C followed by 5 min at 65 °C). This was done in order to improve the efficiency of cell lysis. DNA extractions were conducted in triplicate from the well mixed bulked sludge samples. Extracted DNA was subjected to electrophoresis in a 1% agarose gel in 1 X TAE buffer, and DNA concentration was determined by fluorescence using a PicoGreen® dsDNA quantitation kit (Molecular Probes Inc., Oregon, USA) and a fmax Fluorescence Microplate Reader (Molecular Devices, CA, USA), as described by the manufacturer.

### 2.4 ANAEROCHIP microarray analysis

The 109F and 934R primers (Grosskopf et al., 1998) were used to amplify the 16S rRNA gene of methanogens in the sludge samples by PCR, as described by Franke-Whittle et al. (2009b). Single-stranded Cy5-labeled PCR product was generated using Lambda exonuclease and 500 ng of single-stranded DNA was hybridised on an ANAEROCHIP microarray at 55 °C for 4 h (Franke-Whittle et al., 2009b). Arrays were washed after hybridisation, and a ScanArray Gx microarray scanner (Perkin Elmer, MA, USA) was used to scan hybridised microarray slides. The ScanArray Gx software (Perkin Elmer, MA, USA) was used to analyse fluorescent images, as described by Franke-Whittle et al. (2009b). For all spots, the median foreground and background signals were determined. The signal-to-noise ratio (SNR) for all spots was calculated using the following calculation, as described by Loy et al. (2002):

SNR = [*I*_p_-(*I*_np_–*I*_bnp_)]/*I*_bp_ where *I*_p_ is median intensity of fluorescence of the probe, *I*_np_ is the median intensity of fluorescence of the nonbinding control probe, *I*_bnp_ is the median intensity of fluorescence of the background area around the nonbinding control probe, and *I*_bp_ is the median intensity of fluorescence of the background area around the probe. Signals were treated as positive if a SNR value of ⩾2 was obtained (Loy et al., 2002).

Principal component analysis (PCA) of the total SNR microarray data of the study was conducted, using CANOCO for windows 4.5 (Ter Braak and Šmilauer, 2002).

### 2.5 Real-time quantitative PCR

Real-time quantitative PCR (RT-PCR) was conducted on sludge DNA samples with specific primers targeting the 16S rRNA gene of five different genera of methanogens, determined according to the analysis of the microarray results. The genera investigated were *Methanosaeta*, *Methanosarcina*, *Methanothermobacter*, *Methanoculleus* and *Methanobacterium*. RT-PCR amplifications were conducted using the Sensimix SYBR No-ROX kit (Biotools, Spain) and performed in a Rotor-Gene™ 6000 (Corbett Life Sciences, Sydney, Australia) in 20 μl volumes. Each standard reaction mix contained a final concentration of 1 X Sensimix reaction premix, 100 nM each primer (150 nM for *Methanoculleus* and *Methanobacterium*), 0.04 mg ml^−1^ BSA and distilled water (Franke-Whittle et al., 2009a, Goberna et al., 2010). Sequences of the *Methanosaeta* primers (MS1b and SAE835R), *Methanosarcina* primers (240F and 589R), *Methanothermobacter* primers (410F and 667R), *Methanoculleus* primers (298F and 586R) and *Methanobacterium* primers (fMbium and 748R) are listed in Goberna et al. (2010). One μl sludge DNA (undiluted) was used as the template in each reaction. After an initial denaturation at 95 °C for 5 min, thermal cycling comprised 40 cycles of 20 s at 95 °C, 20 s at 58–65 °C (annealing temperature) and 20 s at 72 °C. The annealing temperatures for the various PCR programs were as follows: 58 °C for *Methanobacterium*, 60 °C for *Methanosaeta*, 61 °C for *Methanothermobacter*, 64 °C for *Methanosarcina* and 65 °C for *Methanoculleus*. Thermal cycling was completed with a melting analysis (65–95 °C, ramp 0.5 °C/min) to check for primer dimer formation and product specificity. Standard curves were constructed with PCR amplified 16S rDNA from pure cultures (*Methanobacterium formicicum* DSMZ 1535*, Methanosaeta concilii* DSMZ 2139*, Methanothermobacter wolfeii* DSMZ 2970, *Methanosarcina barkeri* DSMZ 800 and *Methanoculleus thermophilus* DSMZ 2640) as described in Franke-Whittle et al. (2009a). All standards and samples were run in duplicate.

## 3 Results and discussion

### 3.1 Physical–chemical parameters

Table 2 shows the results of physical–chemical parameter analysis for the two biogas reactors. Neutral, or close to neutral pH values (7.3–7.5) were observed for the duration of the experiment in the Inzing reactor, indicating stable digester conditions. Likewise, the Neustift reactor revealed stable values (7.7–8.0), despite being higher. The values did not vary significantly, despite changes in VFA levels. According to Sandberg and Ahring (1992) and Ward et al. (2008), AD occurs optimally at pH values of 6.8–7.2, and an excessively alkaline pH can potentially result in disintegration of microbial granules and the subsequent failure of the process. The two reactors under investigation nonetheless produced acceptable levels of methane (57.2–65.0%) and were operating stably, despite their higher pH values.

**Table 2:** Physical–chemical parameters of the biogas reactor sludges sampled at different times.

Plant	pH	EC (mS cm^−1^)	TS (%[wt/vol])	VS (%[wt/wt] of TS)	NH_3_–N (mg L^−1^)	NH_4_–N (mg L^−1^)	CH_4_ (%)	CO_2_ (%)	Acetate (mg L^−1^)	Propionate (mg L^−1^)	Isobutyrate (mg L^−1^)	Butyrate (mg L^−1^)	Valerate (mg L^−1^)	Isovalerate (mg L^−1^)
I1	7.3	9.97	3.03	71	15	640	57.2	41.6	25.3	nd	nd	nd	nd	54.0
I2	7.5	11.90	3.58	64	26	720	58.8	40.7	20.8	2.7	159.4	nd	111.7	227.9
I3*	7.4	11.81	3.53	72	19	720	56.8	42.1	1249.6	3910.9	510.0	104.7	647.8	797.9
I4	7.4	12.33	2.91	66	21	800	52.3	47.0	131.2	27.6	nd	nd	nd	24.4
N1*	7.7	28.20	3.67	62	539	3840	64.5	35.4	2281.9	8741.3	650.5	1329.8	1237.2	2623.0
N3	7.9	24.90	3.50	65	636	2880	63.4	35.7	15.7	4.2	nd	nd	nd	nd
N4*	8.0	25.10	5.13	65	721	2960	65.0	34.6	1184.9	4977.5	78.3	712.3	766.8	1189.2

*Note*: nd- not detected.

* High VFA samples.

Conductivity in the two digestion plants varied, with values of 9.97–12.33 mS cm^−1^ obtained for reactor I, and values of 24.90–28.20 mS cm^−1^ for reactor N. Similarly, NH_4_–N and NH_3_–N concentrations varied considerably in the two reactors, as shown in Table 1. Reactor I had significantly lower values (640–800 mg L^−1^ NH_4_–N; 15–26 mg L^−1^ NH_3_–N) than those found in the thermophilic reactor N (2880–3840 mg L^−1^ NH_4_–N; 539–721 mg L^−1^ NH_3_–N). Kayhanian (1999) also showed that the free ammonia nitrogen concentration in a thermophilic reactor can be expected to be six times higher than when compared to a mesophilic reactor at the same pH. Total ammonia nitrogen concentrations of 1.7 g L^−1^ or higher are known to inhibit methanogenesis (McCarty and McKinney, 1961, Koster and Lettinga, 1984, Rajagopal et al., 2013), and archaeal diversity was reported to be affected by concentrations of ammonia exceeding 2000 mg L^−1^ (Garcia et al., 2000, McMahon et al., 2001, Demirel and Scherer, 2008). Total ammonia nitrogen levels in the Neustift reactor sludges were found to be rather high, and thus some inhibition of methanogenesis could have been expected, although it did not occur.

Higher methane concentrations were found in the thermophilic reactor N (63.4–65%). This finding is thus inline with other research, whereby thermophilic processes have been reported to be more efficient than mesophilic processes, and have higher rates of methane production (Cecchi et al., 1991, Griffin et al., 1998, Ho et al., 2013).

VFA levels were found to vary significantly in the two reactors at the different sampling times. Of the four samples collected at different times from the Inzing reactor, only the I3 sludge was found to contain high levels of many VFAs. An acetate level of 1249.6 mg L^−1^ was measured, an amount that according to Hill et al. (1987) would indicate process instability (>13 mM). Elevated propionate levels (3910.9 mg L^−1^) were also detected, resulting in a propionate:acetate ratio far exceeding 1.4, a level considered to indicate process instability (Hill et al., 1987). Butyrate, isobutyrate, isovalerate and valerate levels were, in addition, higher in the I3 sludge sample than in all other Inzing reactor sludges. It is possible that an overloading of the reactor prior to the sampling occurred, however, no information from the plant operators regarding this sampling time is available.

According to VFA analysis, two of the three Neustift reactor sludges analysed were found to contain high VFA levels (N1 and N4). The N1 reactor sludge was found to have the highest level of all VFAs, including acetate and propionate levels (2281.9 mg L^−1^ and 8741.3 mg L^−1^, respectively). VFAs measured in samples collected from the Neustift reactor at other times also contained high VFA levels (data not shown). This would indicate that the Neustift reactor was operating under higher loading rates. Possibly, there was a difference in the OLR of the reactor prior to the collection of the N3 sample, where much lower VFA levels were found. Different researchers have proposed various VFAs as indicators of reactor imbalance, e.g. Kaspar and Wuhrmann (1978) and Nielsen et al. (2007), who investigated reactors treating municipal wastes and manure/industrial waste, respectively, and proposed propionate to be a more appropriate indicator for process instability than acetate. However, according to Hill and Bolte (1989), who investigated reactors treating swine wastes, isoforms of butyrate and valerate are the best indicators of imbalance. In any case, the elevated levels of VFAs in the N1 and N4 sludge samples would suggest process instability and impending digester failure (Nielsen et al., 2007). Methane production rates from the Neustift reactor did not however indicate any problems, and thus it would appear that the microbial communities in the reactor were adapted to a high VFA environment.

The accumulation of VFAs in most situations reflects an imbalance between acid producers (mostly bacteria) and consumers, and is usually associated with a drop in pH and a breakdown of the buffering capacity of the reactor sludge (Ahring, 1995, Akuzawa et al., 2011). The reduction in pH can inhibit the growth of methanogens (Bouallagui et al., 2005, Ye et al., 2013). However, reactor failure, which would be predicted according to the VFA levels in I3, N1 and N4, did not result in the Inzing and Neustift reactors, pH values did not change significantly, and a stable methane production continued. Cow manure was included in the input materials of the two reactors under investigation, and the high bicarbonate and ammonia contents most likely contributed to the buffering of the system (Pind et al., 2003) and prevention of reactor failure. According to Ahring (1995), acetate and propionate concentrations of up to 6000 and 3000 mg L^−1^, respectively, have been shown to cause no inhibition of the AD process in reactors treating manure. Thus, the high acetate concentrations seen in the reactors under investigation may not have been problematic, although the high propionate levels should alleviate concern.

It should also be taken into consideration that all the above mentioned VFA indicator system proposals are based on laboratory scale reactors and research. Our data comes from full scale AD plants. Angelidaki et al. (1993) suggested that various AD plants have their own ‘normal’ levels of VFAs, determined by the composition of the input material entering the reactor, and thus, that it is not feasible to define specific VFA levels which indicate reactor failure. It would appear that these two AD systems had a good specific buffering capacity, and were able to operate stably despite the high VFA levels.

### 3.2 ANAEROCHIP microarray analysis

The ANAEROCHIP microarray, an oligonucleotide array targeting methanogenic genera and species within the orders *Methanobacteriales*, *Methanococcales*, *Methanomicrobiales* and *Methanosarcinales* was used to investigate the diversity of the methanogenic community structure present in the sludges collected from the two reactors at different times. Organisms belonging to these orders are known to dominate and thrive in the anaerobic digester environment (Hori et al., 2006, Demirel and Scherer, 2008, Rastogi et al., 2008). The SNR was calculated for all probes, and those with an SNR ⩾ 2 in one or more samples were used for subsequent analyses.

**Fig. 1:**
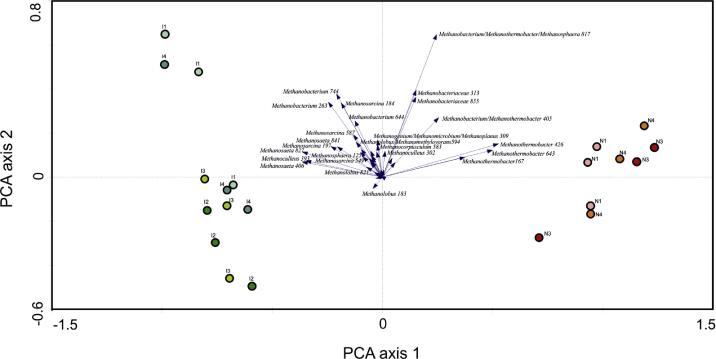
Principal component analysis loading plot depicting the organisms responsible for community differences amongst the Inzing and Neustift reactor sludge samples. Note: The lengths of the arrows indicate the significance for sludge sample differentiation. Arrows of probes point in the direction of samples with above average signal. Reactor I samples are from a mesophilic anaerobic digestion plant in Tirol (I1- 18.05.2009, I2- 05.08.2009, I3^*^- 16.09.2009 and I4 -27.10.2009) and reactor N samples are from a thermophilic anaerobic digestion plant in Tirol (N1^*^ -18.05.2009, N3 -16.09.2009 and N4^*^ -27.10.2009). High VFA samples designated by ^*^.

Fig. 1 shows a principal component analysis (PCA) loading plot, whereby the two first axes explain 87.8% of the variance, the first axis representing 79.5% of the variance, the second axis representing 8.3%. Certain probes (indicated by the arrows) can be seen to be more influential in discriminating the samples. The lengths of the arrows indicate the significance for sludge sample differentiation, and arrows point in the direction of samples with above average signal. Probes with similar arrow directions have high covariance, meaning they tend to occur jointly on the microarrays.

Canonical analysis clearly shows the separate grouping of the mesophilic and thermophilic reactor samples. The Inzing reactor sludge samples were found to be dominated by the genera *Methanosaeta* and *Methanosarcina*. Both genera belong to the order *Methanosarcinales*, acetoclastic methanogens which have been reported to be responsible for approximately 70% of the methane produced in biogas reactors (Jetten et al., 1992, Ahring, 1995). Members of the genus *Methanosaeta* have been reported to dominate in reactors with low levels of NH_3_ and VFAs (Karakashev et al., 2005, Walter et al., 2012), conditions that existed in the I1, I2, and I4 sludges, but not in I3,which contained high VFA levels. Nonetheless, *Methanosaeta* was found in the I3 sample, indicating that the period of high VFAs did not significantly affect the community. *Methanosarcina*, a generalist known to have a high metabolic versatility and able to use acetate, hydrogen, formate, secondary alcohols and methyl compounds as energy sources (Kendall and Boone, 2006), as well as being capable of quickly adapting to changing conditions through higher growth rates (Goberna et al., submitted), was also detected in Inzing reactor samples.

*Methanobacterium*, a hydrogenotrophic methanogen, was detected in reactor I sludges using the microarray, with all four probes yielding signals in the sludges. As expected, the two *Methanobacteriaceae* family probes (313 and 855) and the group probes 405 (*Methanobacterium/Methanothermobacter*) and 817 (*Methanothermobacter/Methanobacterium/Methanosphaera/Methanobrevibacter*) also yielded strong signals. Hybridisations for the probes targeting *Methanoculleus* and *Methanosphaera* were obtained, revealing the digester to host a metabolically diverse methanogenic community, comprised of organisms capable of methane production via various biochemical pathways.

In contrast, samples from the thermophilic reactor were not found to be as diverse, a finding supported by Karakashev et al. (2005). Reactor N sludges were dominated by a community of hydrogenotrophic *Methanothermobacter*. The signals obtained for the *Methanothermobacter* probes were the highest of all genus probes detected (Table 3), indicating both its dominance in the sample as well as a high hybridisation efficiency of these probes. Low signals for 2 of the 5 *Methanoculleus* probes were additionally detected. These findings support the findings of Lee and Zinder (1988) and Petersen and Ahring (1991), whereby high levels of hydrogenotrophic methanogens were found in thermophilic digesters with low acetate concentrations. The hydrogenotrophic dominating methanogen community would indicate that syntrophic relationships between acetate oxidisers and hydrogenotrophic methanogens could be the main pathway for acetate degradation and methanogenesis in the N reactor.

**Table 3:** Signal to noise ratios (SNR) of ANAEROCHIP hybridisations with Inzing and Neustift reactor sludges.

*Note*: Probes with SNR values ≥ 2 are highlighted in dark green. Probes with SNR ≥ 1.5 are highlighted in light green. Probes for *Methanocalculus, Methanocaldococcus, Methanococcoides, Methanomicrobium, Methanobrevibacter smithii, Methanosphaera stadtmanae* and *Methanothermobacter thermoautotrophicus* excluded from table as no hybridisations for any of the probes were detected.

Abbreviations: *Mbact/Mthermbac = Methanobacterium/Methanothermobacter*; *Mlobus/Mmethylovorans = Methanolobus/Methanomethylovorans*; *Mmicrobium/Mgenium/Mplanus *= *Methanomicrobium*/*Methanogenium*/*Methanoplanus*; *Mthermbac/Mbact/Msph/Mbrev = Methanothermobacter/Methanobacterium/Methanosphaera/Methanobrevibacter*.

High VFA samples designated by ∗.

Hybridisation signals were also detected for the group probes targeting Methanobacteriaceae (313 and 855), *Methanomicrobium/Methanogenium/Methanoplanus* (309) and *Methanothermobacter/Methanobacterium/Methanosphaera/Methanobrevibacter* (817). Low SNR values were obtained for two of the *Methanogenium* probes in reactor N samples, explaining the signal for the 309 probe.

Interestingly, *Methanoculleus* was the only genus detected by the ANAEROCHIP microarray in all Inzing and Neustift reactor samples. Schnürer et al. (1999) suggested that its dominance over other hydrogenotrophic methanogens might be related to its tolerance of high salt concentrations. Ammonium levels in reactor N can be considered high (2.9–3.8 g L^−1^), and, according to McCarty and McKinney (1961) and Koster and Lettinga (1984), concentrations above 1.7 g L^−1^ can be inhibitory to some organisms when biogas digesters are not adapted to high ammonium levels. *Methanoculleus* has been reported to dominate in cattle manure fed reactors (Chachkiani et al., 2004, Goberna et al., 2009), despite significant changes in pH and VFAs, supporting the findings of this study.

Table 3 shows the SNRs obtained after hybridisation of the ANAEROCHIP microarray with DNA extracted from AD plant sludges. There did not appear to be any significant differences in the hybridisation signals obtained, or in the fluorescence intensity of signals between the sludges sampled at different times from the same AD plant. This would indicate that the methanogens were not influenced by such fluctuations in VFA levels and reactor conditions, and that the established microbial community was able to withstand some variations in reactor conditions without major changes in methanogen profiles, a finding also found by Wagner et al. (2011). It is also possible, however, that had RNA and not DNA been used in these studies, variations in methanogen numbers may have been detected.

The ANAEROCHIP microarray approach has to be considered ‘semi-quantitative’, and the relative abundance of different microorganisms derived from the microarrays needs to be interpreted with some caution. This is because different probes have different affinities for their targets, and because of the inherent bias involved with PCR amplification. However, a linear correlation between signal intensity of probes and target concentration has been reported by others (Taroncher-Oldenburg et al., 2003, Tiquia et al., 2004). Thus, in order to more accurately investigate methanogen numbers, real-time PCR was applied to genera detected using the microarray.

### 3.3 Real-time PCR

Dominant genera in the sludge sample DNAs according to the results of ANAEROCHIP microarray analysis were chosen as targets for quantification using real-time PCR. Quantitative real-time PCR results largely supported the findings of the microarrays, although were more sensitive, and all genera were detected in all Inzing and Neustift reactor samples. The results and standard curve parameters (slope, intercept, range, *R*^2^ and efficiency) for quantification assays are included in Table 4. Numbers written in italics represent methanogenic genera detected using the ANAEROCHIP microarray, while numbers in bold represent genera not detected using the array.

**Table 4:** Gene copy number g^−1^ sample and standard deviation of *Methanosarcina*, *Methanoculleus*, *Methanobacterium*, *Methanothermobacter*, and *Methanosaeta* as revealed through quantitative real-time PCR of the 16S rRNA gene and standard curve parameters. Numbers written in italics represent methanogenic genera detected using the ANAEROCHIP microarray, while numbers in bold represent genera not detected using the array.

	*Methanosarcina*	*Methanoculleus*	*Methanobacterium*	*Methanothermobacter*	*Methanosaeta*
I1	*6.31E+04 ± 7.01E+02*	*1.07E+04 ± 4.17E+03*	*3.65E+05 ± 9.16E+04*	**1.05E+04 ± 1.16E+02**	*5.37E+06 ± 4.88E+06*
I2	*9.00E+04 ± 7.23E+04*	*6.67E+04 ± 1.05E+05*	*1.53E+05 ± 5.57E+04*	**3.83E+03 ± 2.40E+03**	*8.12E+05 ± 8.17E+05*
I3*	*1.57E+05 ± 1.33E+05*	*3.68E+04 ± 2.64E+04*	*2.75E+05 ± 1.86E+05*	**1.09E+04 ± 1.15E+04**	*1.90E+06 ± 1.36E+06*
I4	*7.33E+04 ± 7.79E+04*	*2.92E+04 ± 2.75E+04*	*4.15E+05 ± 4.25E+05*	**1.57E+04 ± 1.38E+04**	*1.83E+06 ± 2.25E+06*
N1*	**2.56E+04 ± 4.74E+03**	*3.03E+04 ± 8.90E+03*	**5.82E+03 ± 2.63E+02**	*4.94E+06 ± 3.18E+05*	**3.45E+04 ± 4.70E+04**
N3	**1.34E+03 ± 7.21E+02**	*1.27E+05 ± 1.30E+05*	**9.07E+03 ± 4.43E+03**	*9.40E+06 ± 4.62E+06*	**2.68E+03 ± 1.85E+03**
N4*	**1.32E+04 ± 4.01E+03**	*6.18E+03 ± 4.72E+03*	**7.53E+03 ± 2.78E+03**	*9.73E+06 ± 4.68E+06*	**1.23E+04 ± 1.62E+04**
Range^a^	10^2^–10^7^	10^2^–10^6^	10^2^–10^7^	10^2^–10^6^	10^2^–10^7^
*R*^2^	0.99958	0.99956	0.99969	0.99927	0.99983
Slope	−4.268	−4.303	−3.926	−3.518	−3.653
Intercept	44.600	39.013	41.995	32.192	34.643
Efficiency	72%	71%	80%	92%	88%

a Range of standards (gene copies μl^−1^) used for quantifying each genus.

* High VFA samples.

*Methanosaeta* dominated the sludges of reactor I, with gene copy numbers of 8.12 × 10^5^–5.37 × 10^6^ g^−1^ reactor sample detected at different times, despite differing VFA levels. This evidence supports the results from ANAEROCHIP microarray analysis, and indicates that the accumulation of VFAs did not significantly affect the numbers of *Methanosaeta* in the mesophilic reactor, a methanogen known to be sensitive to higher concentrations of VFAs (Griffin et al., 1998).

*Methanosarcina*, as well as the hydrogenotrophic genera *Methanobacterium* and *Methanoculleus,* were also detected in reactor I samples (*Methanosarcina*: 6.31 × 10^4^–1.57 × 10^5^ copies g^−1^ reactor sample, *Methanobacterium*: 1.53 × 10^5^–4.15 × 10^5^ copies g^−1^ reactor sample, and *Methanoculleus*: 1.07 × 10^4^–6.67 × 10^4^ copies g^−1^ reactor sample). *Methanobacterium* and *Methanoculleus* numbers were found to remain stable when VFA levels increased (I3), while numbers of *Methanosarcina* were found to increase in dominance when higher levels of VFAs were detected. Growth of members of the family *Methanosarcinaceae* are favoured under conditions where acetate has accumulated (Demirel and Scherer, 2008), and our findings were thus inline with the findings of others.

Despite not being detected in Inzing reactor sludges using the microarray, *Methanothermobacter* was detected at lower levels than other methanogens (3.83 × 10^3^–1.57 × 10^4^ copies g^−1^ reactor sample) in all four samples investigated. The levels of VFAs in the reactor did not appear to be correlated with *Methanothermobacter* numbers. *Methanothermobacter* has not been frequently reported in mesophilic reactors in the literature (Ahring, 1995).

The thermophilic Neustift reactor was found to be dominated by *Methanothermobacter*, with numbers of 4.94 × 10^6^–9.73 × 10^6^ copies g^−1^ reactor sample. Hori et al. (2006) found that species of *Methanothermobacter* began to dominate in a thermophilic reactor when VFA levels increased, and in particular, propionate. As was found in reactor I, the levels of VFAs in the reactor did not appear to be correlated with *Methanothermobacter* numbers, and our results did not indicate an increasing dominance of the genus in the N1 and N4 samples which contained high VFAs.

*Methanosaeta* was detected by real-time PCR in reactor N samples (2.68 × 10^3^–3.45 × 10^4^ copies g^−1^ reactor sample), despite not being detected using the ANAEROCHIP array. *Methanosarcina* and *Methanobacterium* were also detected at lower levels in reactor N samples (up to 2.56 × 10^4^ and 9.07 × 10^3^copies g^−1^ reactor sample, respectively), despite not having been detected using the microarray. This difference may be attributable to the fact that general archaeal primers were used to amplify DNA from the sludge samples for microarray analysis, while genus specific primers were used in real-time PCR analysis. It is possible that a bias existed with the primers towards the more dominant methanogens, such that the less dominant organisms were not amplified enough to be detected using the microrarray. It is also possible that the cell numbers of *Methanosarcina* and *Methanobacterium* were below the detection limit of the array (∼0.4 pg DNA, Franke-Whittle et al., 2009b). *Methanosarcina* was found to increase in dominance when higher levels of VFAs were present, as was found in reactor I. The numbers of *Methanobacterium* did not appear to be affected by VFA concentration. *Methanobacterium* is a genus containing hydrogenotrophic methanogens. Studies conducted by Griffin et al. (1998) concluded that species of the genus *Methanobacterium* were the syntrophic partners of acetate oxidisers in a thermophilic digester treating municipal solid waste and biosolids, and dominated by *Methanobacteriaceae*. Although *Methanobacterium* did not dominate in samples from either reactor, species of the genus have been reported by others to be dominant in anaerobic digesters (Godon et al., 1997, Leclerc et al., 2001).

Similar copy numbers of *Methanoculleus* were also detected in all Neustift reactor samples (3.03 × 10^4^–1.27 × 10^5^ copies g^−1^ reactor sample). According to Goberna et al. (2009), *Methanoculleus* remained the dominant methanogen in a thermophilic reactor, despite significant changes in pH and VFAs. In contrast, Hori et al. (2006) showed that *Methanoculleus* numbers in a thermophilic anaerobic digester declined during the accumulation of VFAs.

### 3.4 Correlation analyses

To detect correlations between the various physical–chemical parameters and methanogenic genera in the two reactors, data were subjected to analysis using the PASW-SPSS 17.0 software. Results are presented in Table 5, Table 6. Significant positive correlations (*p* < 0.01) are shaded in dark green, those with a *p* < 0.05 in light green. Negative correlations (*p* < 0.05) are shaded in yellow. Correlation analysis for the reactor I reactor indicated significant correlations between *Methanosarcina* and all VFAs (Table 5). The higher growth rates at high acetate levels of species of *Methanosarcina* when compared to *Methanosaeta,* have been reported by others (Zinder, 1993, Conrad and Klose, 2006, Demirel and Scherer, 2008, Walter et al., 2012), and thus the correlation was not surprising. Similarly, the majority of VFAs were positively correlated with each other, as expected. Interestingly, apart from significant correlations (*p* < 0.05) between NH_3_–N with pH and NH_3_–N with *Methanoculleus*, there were no other significant correlations of parameters in the reactor with each other, or with the methanogens. These correlations are in contrast with the findings of Niu et al. (2014), who reported a distinct rise in VFA accumulation at high NH_3_–N levels, as well as the dominance of *Methanoculleus* in an AD reactor prior to the addition of high amounts of NH_3_–N, and the absence of the genus after inhibiting ammonia conditions had been created*.*

**Table 5:** Correlation analysis of physical–chemical parameters and methanogenic genera present in Inzing reactor sludges.

*Note*: Significant positive correlations (*p* < 0.01) are highlighted in dark green, those with a *p* < 0.05 in light green.

Abbreviations: Msarcina- *Methanosarcina*; Mculleus*- Methanoculleus*; Mbacter*- Methanobacterium*; Mtherbact*- Methanothermobacter*; Msaeta*-Methanosaeta*.

^c^Not able to be determined.

**Table 6:** Correlation analysis of physical–chemical parameters and methanogenic genera present in Neustift reactor sludges.

*Note*: Positive correlations with a *p* < 0.05 are highlighted in light green, and negative correlations with a *p* < 0.05 are highlighted in yellow.

Abbreviations: Msarcina- *Methanosarcina*; Mculleus*- Methanoculleus*; Mbacter*- Methanobacterium*; Mtherbact*- Methanothermobacter*; Msaeta*-Methanosaeta.*

Different significant correlations were detected in the reactor N sludges (Table 6). Certain VFAs were positively correlated with each other, as seen in reactor I. However, in contrast to reactor I, *Methanosarcina* was not significantly positively correlated with all VFAs, rather, only with acetate, butyrate and isovalerate. *Methanobacterium*, a hydrogenotrophic methanogen, was significantly negatively correlated with the same VFA, as well as with *Methanosarcina. Methanothermobacter*, which was dominant in the thermophilic reactor, only correlated significantly positively with the VS.

## 4 Conclusions

In this study, the hypothesis that changes in VFA levels of anaerobic digester plants affect the indigenenous methanogenic communities was investigated. Two different anaerobic digesters (one operating under mesophilic conditions and the other under thermophilic conditions) which experienced variation in VFA levels were monitored in terms of physical and chemical analyses. In addition, methanogenic communities were investigated using DNA based tools.

The methanogenic community composition was similar in the mesophilic and thermophilic reactors, however, the structure was different. According to microarray analysis, the mesophilic reactor was shown to be dominated by a more diverse community, comprising *Methanosarcina, Methanoculleus, Methanobacterium* and *Methanosaeta*, while the thermophilic community was dominated by *Methanothermobacter*. Real-time PCR results confirmed the findings of the microarrays, but were quantitative and more sensitive, indicating a greater diversity.

Our findings support those obtained not only in other biogas reactors, but also in other environments such as upland pasture rhizosphere soil (Nicol et al., 2004), where acidification appeared to have no significant effects on the indigenous community of methanogenic archaea. It would appear that the two AD systems investigated had a good buffering capacity, and that the microbial communities were able to withstand changes in VFA concentrations.
